# 

**Published:** 1921-07-09

**Authors:** 


### A Hospital Family.

SIR FREDERICK CHANCE has been elected President of the Home for Incurables, Carlisle, in succession to the late Dr. Barnes. Associated for thirty-eight years with the institution, Sir Frederick has been its Honorary Secretary. In this office he is now succeeded by his son, Mr. R. C. Chance.

### National Baby Week.

As we go to press the activities in connection with this annual function are proceeding. In London the second English-speaking Conference is being held at the Central Hall, Westminster, the inaugural address being delivered by Viscount Astor. The subjects of the various papers cover a wide ground, and we propose to deal with them inclusively in our next issue. The Child Welfare Exhibition in connection with the Conference remains open till Saturday, July 9. The London Federation of Infant Welfare Centres has organised visits during the week to a large number of such institutions.

### THE BRITISH HOSPITALS ASSOCIATION.

### Annual Meeting.

### The Inquiry System at Cardiff.

THE Board of Management of King Edward VII. Hospital, Cardiff, which has an overdraft of £35,000, has adopted the scheme of the Finance Committee whereby all patients who contribute to organisations at the minimum rate of a penny per week shall be exempt from personal payments toward their maintenance in hospital, but that all others shall be required to contribute in proportion to their means. On receipt of a ticket of application for admission inquiry will be made personally or by letter as to how much the patient is able to pay. The amount will then be entered on the records, and the money collected as a matter of routine when the patient is admitted. It may be found expedient to differentiate by colour or otherwise in the tickets issued, and as every patient will be required to furnish information how he contributed to the hospital, if at all, questions asking this information will be printed on future issues of tickets. By these means the hospital hopes to secure new income from those 55 per cent. of patients who at present do not contribute to the funds in any way. At the end of three months the scheme is to be reported upon to the Board, so that its value may be assessed.

### Support through Local Committees.

### Encouraging Prospects at Shrewsbury.

"Long maintained by the subscriptions and donations of a comparatively small number of persons, the Royal Salop Infirmary now appeals for help to the entire county, employers and employed." This constitutes both an appeal and a hope expressed by the Treasurer, Lord Berwick, at the recent annual general meeting of the Royal Salop Infirmary, Shrewsbury. In a review of the year's work his lordship said at the time of his election the Infirmary, like other hospitals throughout the country, found itself at the turning of two roads, and the Board of Directors had the responsibility of deciding which of the ways they should take—the old road, which was the open road, or a road that led through what might be termed a toll-gate, that of paying patients. The latter they were unwilling to take because they believed it would eventually lead them to an institution State-managed, State-controlled, with all the inconvenience of officialdom. They decided to maintain the voluntary system and call to their aid those classes of the community who most needed the Infirmary. A scheme had been inaugurated under which local committees were being formed in municipal boroughs and urban district councils throughout the county of Salop and in parts of Montgomeryshire and Radnorshire. The scheme, thanks to the untiring energy and enthusiasm of the Secretary, had met with very encouraging support, yearly contributions of £1,500 having been already promised. One of their directors, Mr. George Fryer, had obtained from the miners of Shropshire the promise of a voluntary levy of 1d. per week, and 2d. in some cases, as soon as conditions became normal; the railwaymen had also promised substantial subport. When the Secretary had visited the whole district it was hoped in this way their annual income would be increased by about £5,000. This year they had relied more on donations than subscriptions and he was happy to say donations amounted to over £5,000 in excess of the average. From church and chapel collections they had received £786, as against £696. The directors appealed to all churches and chapels to give the Infirmary at least one offertory during the year. Subscriptions during the year had increased by £600, and amounted to £3,359, for which they were extremely grateful. The total ordinary income amounted to £16,496, against which their expenditure was about £18,600, leaving, in spite of increased revenue, a deficit of £2,104. The Infirmary had been very full throughout the year, the number, both in in-patients and outpatients, having increased. In conclusion Lord Berwick acknowledged the handsome gift of £9,000 from the Shropshire War Memorial Fund, with which it was proposed to build a children's wing. As this would cost at least £15,000 they must wait until conditions were more favourable before work was started.

### A Successful Hospital Fete.

Organisers of the Gravesend Hospital Fête, held on June 29, deserve hearty congratulations on the magnificent result of six months' united endeavour to bring the undertaking to a successful issue. The favourable weather, which brought thousands of visitors, and the gracious act of Princess Mary in visiting the Fête for the purpose of receiving purses, were also powerful factors in their success, and the financial result is already well over £5,000. Of this, £1,000 was contributed in the form of a stock certificate for that amount, which was presented by Captain H. Davis, Ruler of Pilots, on behalf of the Trinity House pilots stationed at Gravesend, for the purpose of endowing a "Pilots' Bed." Money is badly needed by the hospital to pay off a debt of £2,000, and another £1,000 for exterior and interior renovation, and these needs will be now supplied from the proceeds of the Fête. During the war the wards were among the first to open their doors to the wounded, and among other benefits more than 5,000 soldiers were x-rayed there. In 1913 the daily average of patients was thirty-five; now it is sixty; and the cost has gone up from £3,500 to £10,500. Twelve new beds have been added during the last few years, which accounts for some of the extra expenditure, and the work has increased in every direction.

### Personalia.

Mr. Herbert Gill, Chairman of the Executive Committee of the Bradford Hospital and Convalescent Fund, has now completed thirty-three years' continuous work for the hospitals of the city. Before he became its chairman in 1915 he was successively a member and honorary secretary. His organising ability has been recognised by his appointment this year as President of the Yorkshire Voluntary Charitable Association. These posts by no means exhaust the connections which he has with different charities, and it would be difficult to find a fuller record than his long and active period of hospital service.

### Passing Events and Topics.

### "Touting" by Health Visitors.

A Walsall Town Councillor recently made the strange allegation that the medical officer of health was responsible for what he considered canvassing and touting by health visitors in the course of their duties, and the Councillor said a repetition of the offence would be followed by the matter being brought to the notice of the British Medical Council. Dr. Harry Shore has replied to the strange complaint in a report he has submitted to the Corporation, pointing out that at homes which they visit the health visitors advise, when they think desirable, mothers to bring their babies to the Welfare Centre. With the increasing unemployment, presumably these occasions will also increase. They have no object to serve in these cases other than their welfare, and the efforts are appreciated and bear fruit.

### Training Pupil Midwives.

In connection with the training of pupil midwives at Hammersmith Council's Maternity Home, the matron has had a communication from the C.M.B. with reference to her recent application for reapproval as a teacher of midwifery. The Board desires that arrangements should be made whereby the pupils at the Home will get experience in the work in the district, in addition to the Home's training, and suggests that for this purpose arrangements could probably be made with midwives practising in the vicinity. The Maternity Committee of the Borough Council agrees that facilities for outside training of pupils should be provided, and that this could best be secured by the trained staff at the Home being authorised to accept and attend cases at the homes of the patients outside. The existing staff of midwives is not sufficient to permit of this, and the Committee therefore suggests the engagement of an additional midwife, and that the whole of the midwives should in turn attend cases outside.

### Football Profits for Hospitals.

The Keighley Victoria Hospital has received through the Friendly Societies Gala Committee close on £300, the profits on the football matches for the Keighley Charity Cup—a record sum. The Gala Committee are endeavouring to break all previous records this year by making their quota to the hospital reach over £1,000.

### Pay from Patients.

At the last meeting of the Willesden Urban District Council the Health Committee was asked to consider the advisability of "making a charge for medical treatment received." The Health Committee has since gone into the subject, but, though it has rejected a resolution to make a charge, it has at the same time asked the member who proposed the charge to prepare a scale of charges which can be considered by the Committee.

### Viscount Knutsford's Latest.

A witty impromptu by Viscount Knutsford was immensely appreciated at the Annual Meeting of the Medical Research Defence Society. Dr. Dale had given a paper in which he modestly ignored his own work and praised that of Dr. Hill. "It seems to me," remarked Viscount Knutsford, "that this is an up-Hill and down-Dale affair!" Lord Knutsford added that the more he knows of these scientific men the more he appreciates their humility and wants to protect them against the unjust slanders that are constantly spread by ignorant people.

### St. Mary's Hospital Musical and Dramatic Society.

A concert was held on Wednesday, June 29, in the Library. In addition to orchestral pieces and vocal and instrumental numbers, the programme included a revue entitled "Hall—O.P.D.!" In this all the typical characters of the out-patient department were depicted—the chiefs, the students, the nurses, and last but not least, the patients. Amongst these last, the deaf man and the "loquacious lady with her itching infant" were very cleverly portrayed.

### £8,500 from a Festival Dinner.

The festival dinner held in aid of the Royal Hospital and Home for Incurables, Putney, at Grocers' Hall, on June 9, with Sir James Carmichael, K.B.E., in the chair, realised £8,500. The Chairman contributed £1,000 of this.

### Mountain Climbing by Motor-Car.

A wonderful mountain excursion, made by motor-car in eight daily stages, is now running from Nice to Ballon d'Alsace, a distance of 621 miles, through the French Alps, the Jura, and the Vosges, penetrating by way of some of the highest passes to wild and rugged mountains. The tour is organised by the Paris, Lyons and Mediterranean Railway, whose headquarters are at 179 Piccadilly.

### Willesden Hospital Designs.

What should be a most interesting exhibition for hospital architects and managers is to be held at the galleries of the Royal Institute of British Architects, Conduit Street, W. 1, from July 11 to 18 inclusive, where will be shown seven competitive designs for the extension of the Willesden Hospital as Willesden's War Memorial, including those of the authors of the prize plans, Messrs. Greenaway & Newberry, A.R.I.B.A., of Parliament Mansions, Victoria Street, Westminster. The plans provide for a hospital of 120 beds, with a possible extension to 240, on an island site of 6½ acres, the buildings to be on the pavilion system.

### Holidays for Working Women.

The Committee of the Women's Holiday Fund, 76 Denison House, 296 Vauxhall Bridge Road, S.W., once again appeals for help from the public to give the tired working women a holiday with the refreshment of fresh air and sea breezes. The mother is allowed to bring her baby, and the cost for the two for a fortnight, including railway fare, is £3 10s. The Women's Holiday Fund has just opened a small Home of its own at St. Leonard's, with a trained matron in charge.

### Art as a Solace in the Ward.

The Arts League of Service (1 Robert Street, W.C. 2), whose aim is "to bring Art into everyday life," recently gave an entertainment in the Prince Francis Ward of the Middlesex Hospital. It was easy to see how much the patients liked the fine old folk songs and dainty Elizabethan ditties, sung and acted in picturesque costumes (designed by artists), and the graceful Greek "expressionist" dancing that the A.L.S. gave them. "It was so restful and happy; just what we could enjoy," was their verdict. The entertainment was one of a weekly series arranged by the Dowager Lady Brassey for the patients in the Middlesex Hospital. Its success suggests new possibilities for Art, whose place in national life is now being greatly discussed. Why should it not take its definite place in the work of the hospital?

### Hospital Sunday, 1921.

The first list of amounts received at the Mansion House includes donations of £5,000 from Lady Strathcona and Mount Royal, and £2,500 from Mr. and Mrs. H. G. Tetley and children; also an anonymous donation of £1,000. From the churches the following has been received: St. Anne's, Wandsworth £183; St. Peter's, Cranley Gardens, £165; St. Giles', Cripplegate, £163; Union Church, Mill Hill, £139; Westminster Chapel, £144; Temple Church, £148; All Saints', Knightsbridge, £128; St. Andrew's, Lambeth, £121; Chapels Royal, £117; Wanstead churches, £115; St. Alphage and St. Mary, Aldermanbury, £110; Hornchurch Parish Church, £110; Whitechapel Primitive Methodist Mission, £105. The total to date is £50,000.

July 9, 1921] [Price Twopence

### The Hospital

**RATES OF SUBSCRIPTION** (post free, payable in advance).

**Three months, 3/3; six months, 6/6; twelve months, 13/-. (Should the cost of printing and paper as well as increased postage, necessitate alteration of these rates, the period paid for will be reduced proportionately on unexpired subscriptions.) THE HOSPITAL "Index" to Volume LXIX., October 1920 to March 1921, is now ready, price 1/1 per copy, post free. Address The Manager, THE HOSPITAL, "The Hospital" Building, 28/29 Southampton Street, Strand, London, W.C. 2.**

